# CAREMOBI: AN APP CONNECTING CAREGIVERS, ADULT DAY CENTERS, AND HEALTHCARE PROVIDERS CARING FOR PEOPLE WITH DEMENTIA

**DOI:** 10.1093/geroni/igac059.684

**Published:** 2022-12-20

**Authors:** Tina Sadarangani, Jonelle Boafo, Jie Zhong

**Affiliations:** New York University, New York, New York, United States; Rory Meyers College of Nursing, New York, New York, United States; New York University, New York, New York, United States

## Abstract

Fragmented communication between care partners, health care providers, and adult day centers around the needs of persons living with dementia (PLWD) contributes to avoidable health care utilization. CareMOBI, is a user-centered mobile application that streamlines information exchange around datapoints relevant to the care of PLWD. CareMOBI facilitates team-based communication around emerging clinical problems by uniquely integrating the knowledge and day-to-day observations of adult day center staff and care partners. We present a stakeholder engaged approach to iteratively designing and validating an initial prototype. Through interviews and focus groups with adult day center staff (n=31), primary care providers (n=22), and family caregivers (n=13) we identified barriers to communication across settings. We then visually mapped the domains of a future app which we subsequently validated and refined with end-users (n=25). We present our current prototype which synthesizes these findings and addresses key barriers to information exchange across community settings serving PLWD.

